# Incorporating active commuting into daily life: a narrative review of e-bikes’ impact on health and urban air quality

**DOI:** 10.3389/fspor.2025.1662076

**Published:** 2025-11-10

**Authors:** Alessandro Sampieri, Antonio Paoli

**Affiliations:** Department of Biomedical Sciences, University of Padua, Padua, Italy

**Keywords:** e-bike, active commuting, physical activity, sedentary behavior, air pollution, urban mobility

## Abstract

In the face of rising physical inactivity, sedentary lifestyles, and worsening air quality, innovative solutions that promote individual well-being, public health, and urban sustainability are urgently needed. Active commuting (AC) and particularly e-bikes offer a promising and scalable intervention, particularly in Europe, where millions of workers commute daily, and the transport sector remains a leading source of pollution. This narrative review was conducted by searching peer-reviewed articles and institutional reports on scientific databases until December 2024, focusing primarily on European studies. This review examined the impact of AC and e-bikes on physical activity, health, air quality, urban mobility, and technological innovations. Findings reveal that AC and e-bikes represent a viable alternative to integrate low- to moderate-intensity physical activity into daily routines, while mitigating the health risks associated with prolonged sitting. Evidence also showed that e-bikes contribute to reduced CO₂ emissions and improved urban mobility. However, adoption rates across Europe remain relatively low and heterogeneous, making it crucial to understand the individual, and social factors influencing their use. Beyond their physical and environmental benefits, e-bikes are increasingly embedded within intelligent transport systems, featuring IoT connectivity, real-time monitoring, and user-centered design that enhance safety, and user engagement. This review highlights the role of e-bikes as a bridge between public health, urban planning, and digital innovation, providing actionable insights for policymakers committed to promoting active lifestyles and building more inclusive, resilient, and sustainable cities.

## Introduction

The global increase in air pollution poses significant challenges to public health, contributing to various cancers and respiratory and cardiovascular diseases (1, 2). Concurrently, modern sedentary lifestyles have exacerbated the prevalence of chronic conditions such as obesity, diabetes, and hypertension (3). In this context, active commuting (AC) has been widely recognized as a dual-benefit strategy that improves health through physical activity while reducing air pollution (4). Research on active travel is extensive, but much of the existing evidence concerns general mobility purposes, which include, for example, leisure or shopping. Commuting, however, is a distinct domain shaped by unique determinants, including time constraints, distance, and workplace-related factors. For this reason, it requires a dedicated discussion to better understand not only its specific health, environmental, and organizational implications but also the barriers that may hinder its adoption.

Indeed, although the potential health and environmental advantages of AC, conventional cycling or walking as commuting strategies face substantial barriers, such as long travel distances and physical exertion, limiting the adoption rate (5, 6). Electric bicycles (e-bikes), on the other hand, offer a viable alternative combining motorized assistance with physical activity, ensuring higher speed with less effort and making AC more accessible to broader segments of the population (7). However, the integration of e-bikes into AC policies remains underexplored, with the existing literature often fragmented across health, environmental, and transport fields. Thus, a more cohesive perspective is required to clarify the potential contribution of e-bikes to AC.

The European context provides a compelling case for exploring the role of AC and e-bikes. On the one hand, ∼75% of European cities still face persistently high levels of urban air pollution and traffic congestion, with inhabitants frequently exposed to pollutant concentrations exceeding WHO health-based guidelines (8). Thus, the commitment of the European countries to achieve sustainability goals creates a fertile ground for interventions and studies on AC strategies. On the other hand, compared to regions such as China, where the market of e-bikes is more advanced, European countries continue to show relatively low and heterogeneous levels of use (9, 10). This highlights the importance of focusing on the European context, where research is needed not only to assess health and environmental benefits but also to identify barriers, regional disparities, and effective strategies to inform policies and practice.

Thus, after synthesizing the current evidence on physical inactivity, sedentary behavior, and air pollution as key public health and environmental challenges in Europe, this review aims to: a) examine the health, environmental, and urban mobility benefits of AC, with a particular focus on e-bikes; b) highlight the main barriers and concerns limiting the adoption of e-bikes for commuting, providing actionable insights and best practices to overcome them, and d) explore the technological innovations that can enhance safety, accessibility, and enjoyment.

To these scopes, a literature search was conducted by searching Google Scholar and PubMed databases up to 31st December 2024 without any restriction on the year of publication. A combination of search terms was used, including but not limited to: “e-bike OR electric bicycle OR pedelec”; “active commuting OR active transportation”; “physical activity OR exercise”; “air pollution”; “Europe”; “intelligent transportation”; “sensors”. Further studies were retrieved by screening the reference lists of the selected articles. We included peer-reviewed publications only in the English language and official reports from the most authoritative institutions (e.g., World Health Organization, European Environmental Agency). Published conference abstracts or non-full-text articles available were excluded. For the sections related to e-bikes, studies concerning fully electric bikes (without pedal assistance) or non-commuting recreational uses were excluded.

Building on this search strategy, this review highlights the current health problems related to physical inactivity and sedentary behavior (Section 1.1), and air pollution (Section 1.2); examines the impact of AC on health, workplace wellness, and air quality (Sections 2.1 and 2.2); provides details on the main barriers to AC and the current rate of adoption in Europe (Section 2.3). The review then highlights the unique advantages of e-bikes in the context of AC (Section 3), including their barriers to adoption and best practices and strategies to limit them (Section 3.1).

### 1.1 Physical inactivity and sedentary behavior

Physical inactivity and sedentary behavior represent two different challenges. Sedentary behavior is defined as “any waking behavior characterized by an energy expenditure ≤1.5 metabolic equivalents (METs), while in a sitting, reclining or lying posture” (11), where a MET is a measure of the resting metabolic rate, i.e., the amount of oxygen consumed at rest. Sedentary behavior is common across daily life and includes activities such as watching television, sitting at work, and commuting, where energy expenditure is relatively low. In contrast, physical inactivity accounts for the failure to meet the World Health Organization's (WHO) physical activity guidelines, which recommend 150–300 min of moderate-intensity (i.e., 3 ≤ MET ≤ 5.9), or 75–150 min of vigorous-intensity (i.e., MET ≥ 6) physical activity, or some equivalent combination of moderate- and vigorous-intensity physical activity per week (12).

In Europe, one out of three adults do not engage in sufficient physical activity, and approximately 50% never exercise or participate in sports (13). The widespread inactivity led to a considerable socioeconomic impact, as it has been estimated that in 2012, Europe spent approximately €9.2 billion in direct healthcare due to physical inactivity (14). Moreover, physical inactivity is responsible for 6%–10% of noncommunicable diseases (e.g., coronary diseases, type 2 diabetes, cancers), which account for 74% of all deaths globally (15, 16). Several internal and external factors contribute to physical inactivity. According to the Centers for Disease Control and Prevention, the most critical barriers in adult life are insufficient time, lack of energy, and motivation (17), as well as the absence of exercise facilities and social support (18). Environmental factors, such as traffic and air pollution, disengage physical activity and mitigate its health benefits by increasing pollutant inhalation while exercising (19–21), a process caused by increased expansion of the lungs and increased frequency of breath during physical activity.

Alongside physical inactivity, sedentary behavior represents a significant risk factor associated with clinical conditions, including type II diabetes and lung and colon cancers (22). A recent meta-analysis revealed a strong correlation between the time spent sitting and increased risk for all causes and cardiovascular mortality (3). Specifically, the mortality rate increases by 2% for each hour spent sitting and reaches 8% per hour when the time spent sitting exceeds eight consecutive hours (23). Nevertheless, the number of Europeans who spend more than 4.30 h sitting increased from 49.3% in 2002 to 54.5% in 2017 (24), exacerbated by the COVID-19 pandemic, which further heightened worldwide daily sedentary time by 135.0 on average (25).

Sedentary behavior and physical inactivity include different domains, such as work, home, transportation, and leisure. This distinction is important since researchers have shown that while leisure-time exercise has increased, physical activity related to work, home, and commuting has significantly declined (7). Technological advancements such as computers and wireless communication devices have minimized the physical effort required to perform different daily tasks, with work-related activity decreasing energy expenditure by over 100 calories over the past 50 years (26). Furthermore, urbanization and car-oriented infrastructure contributed to the spread of motorized vehicles and cars for commuting, increasing the opportunities for sedentary behavior (27). This is linked to a greater risk of adverse health outcomes, such as overweight and obesity (28), with every extra hour spent in a car daily increasing the risk of obesity by 6% (29).

### 1.2 Air pollution

The rapid increase in motorized commuting journeys contributed to congestion, air pollution, greenhouse gas emissions, car accidents, and noise. Air pollution, as defined by the WHO, is the contamination of indoor or outdoor environments by any physical, chemical, or biological agents, and represents the leading environmental risk factor for health (30). Specifically, in 2021, the European Environment Agency estimated that exposure to fine particulate matter (PM) and nitrogen dioxide concentrations led to 253,000 and 52,000 deaths, respectively (31). Additionally, in 2022, 96% of the urban population in Europe was exposed to fine PM levels exceeding the health-based guidelines set by the WHO, with Central-Eastern Europe and Italy reporting the highest concentration (32). Despite overall improvements in air quality, current European Union standards are still not met across Europe (31). The primary sources of air pollution are industrial activities, energy production and heating plants, and car traffic. For the purpose of this review, we focus on transportation-related pollution.

Urban commuting alone contributes up to a quarter of the global CO_2_ in European countries (33) and accounts for up to 66% of PM_2.5_ levels (34). Cars, together with vans, are the main contributors to transport pollution in Europe, accounting for about 13% of total greenhouse gas emissions (35). Ineffective urban mobility is associated with increased local pollution levels, low air quality, and poor quality of life. Indeed, drivers, commuters, and individuals living in proximity to main roads exhibit increased rates of morbidity and mortality, particularly from cardiovascular events (36, 37). Moreover, a longer exposure time to traffic congestion is associated with higher systolic and diastolic blood pressure (38), increasing the risk of hypertension. Overall, the heightened exposure to air pollution is linked to a range of health conditions, including respiratory diseases and cardiovascular events (1, 39), type 2 diabetes (40), and cancer (2).

In addition to the environmental impact caused by the direct use of motorized vehicles, the ecological footprint of their production should be considered: 80 million cars are built annually, resulting in car companies contributing to 4% of total CO_2_ emissions (27).

This scenario underscores the urgent need for European policymakers who are engaged in an ongoing search for an optimal approach to deal with economic, social, and ecological crises and the rapid acceleration of climate change (41). In fact, United Nations member states are committed to fulfilling the accords to implement the Sustainable Development Goal of the 2030 Agenda. Specifically, for the purposes of this review, the following objectives are mentioned: (a) promoting health and well-being, (b) making cities sustainable, and (c) taking action against climate change. Moreover, the European Union is committed to reducing the transport emissions by 90% by 2050, and initiatives to reach this aim include not only the decarbonization of motorized vehicles (e.g., by electrifying vehicles) but also promoting active mobility (42).

## 2 Active commuting to promote physical activity and reduce air pollution: evidence related to health, air quality, and urban mobility

AC is a subcategory of active transportation, which refers to any form of human-powered transportation. While active transportation encompasses a variety of activities, including walking and cycling for various purposes (e.g., shopping), AC specifically focuses on daily travel between home and work. Public policies look with interest at AC because it promotes physical activity among workers while alleviating urban pressures and greenhouse gas emissions.

### 2.1 Health impact of AC

The health effect of AC relies mainly on the increase in time spent “on movement”. Overall, regular physical activity can help individuals manage body mass due to increased energy expenditure during exercise, strengthen muscles, and improve mental health and the ability to perform daily tasks (43, 44). Those who meet international physical activity guidelines have a 20%–30% reduced risk of premature death and chronic diseases (45). Moreover, physical activity is associated with better mental health, including improved self-esteem and mood and reduced depressive symptoms, stress, and anxiety (46). Additionally, high levels of moderate-intensity physical activity can cancel the increased mortality risk associated with prolonged sedentary behavior (47).

Although the benefits mentioned above are common knowledge, high levels of physical inactivity in Europe persist (*see 1.1 section*). Thus, it is necessary to create and reinforce strategies to increase adherence to physical exercise, such as promoting activities in the workplace or during commuting. AC transforms the necessary task of commuting into an opportunity for regular exercise without requiring additional time commitments for workers with busy schedules. Indeed, AC has gained popularity as a feasible way to increase physical activity and thus improve health (48, 49).

A meta-analysis that mainly included studies in Europe (particularly in Scandinavian countries) revealed that AC has a protective effect on cardiovascular health (50). Moreover, a recent study conducted in a British sample revealed that AC was associated with a reduced risk of all-cause mortality, cardiovascular diseases, and cancer incidence (51). Furthermore, research based on statistics in the Netherlands highlights that switching from a car to a bicycle for daily journeys could increase life expectancy by 3–14 months (52). Additionally, a randomized control trial performed in Denmark revealed that people who shifted to AC by bike for 6 months improved cardiometabolic health (i.e., increased peripheral insulin sensitivity and cardiorespiratory fitness) (53). Overall, compared with car-only users, individuals who actively commute present a reduced body mass index (BMI) and body fat percentage, variables associated with a greater risk of cardiometabolic diseases (54). Although AC may increase exposure to pollution inhalation, traffic injuries, and noise, research has shown that the overall health benefits of active transportation outweigh the risks associated with environmental hazards (e.g., traffic injuries) (55, 56), except in areas with extremely high levels of PM_2.5_ (56).

The advantages of AC extend to psychological outcomes and working well-being. A British survey revealed that active commuters reported higher levels of psychological well-being compared to those relying on cars or public transport, and a switch from passive to active transportation was linked to improved overall well-being (57). Moreover, a research study on Dutch workers found that those who commute via bikes had, on average, 1.3 fewer sickness absences annually (58), which means a gain of approximately 5 billion euros per year for employers around the European Union (59). Similar results were shown in a randomized control trial performed in Austrian commuters, where individuals who engaged in AC for twelve months by cycling reported greater health-related quality of life and those who commute via both public transportation and walking reported fewer days of sick leave (60). Furthermore, AC is positively associated with more productive organizational behavior (61), an important aspect that companies should take into account when promoting employee well-being. Overall, workers who actively commute report lower levels of depression and mental health diseases and higher levels of life satisfaction (62, 63). Thus, companies should consider these aspects and encourage AC among workers.

### 2.2 Air pollution management and urban mobility

The promotion of AC helps manage pollution and alleviate urban traffic stress. A study across seven European cities found that shifting from cars to bicycles reduced daily CO_2_ emissions by 3.2 kg per person, with a potential 10% reduction in overall emissions if 10% of citizens adopt active transport (64). In Porto, Portugal, modeling studies have suggested that replacing just 5% to 20% of motorized vehicle trips with walking or bicycling could save an average of 168.2 and 336.4 kg of CO_2_ per commuter each year if motorized transport is replaced with walking and 84.1–252.3 kg with cycling (65). Beyond European countries, research in New Zealand estimated that switching short car trips to active transportation may reduce greenhouse gas emissions by approximately 194 kg of CO_2_ annually (66). Similar findings were observed during car-free days, such as CicLAvia in Los Angeles, where road closures led to reductions in PM_2.5_ levels (67).

AC also improves urban mobility. Compared with motorized transportation, bicycles take up much less space on the road, improving urban traffic, and require significantly less space for parking compared to cars. This saved urban space can be reallocated to green areas or other community-friendly infrastructure, further enhancing environmental quality (5, 37). Given the strong impact of air pollution on public health and the multiple environmental and mobility-related benefits of AC, many European cities are increasingly redesigning their urban spaces to facilitate AC and align with climate change and air quality policies.

For example, several cities have implemented clean air zones that restrict or ban the access of motorized vehicles during specific hours, as seen in London and Milan. In addition, congestion charging schemes are becoming more widespread, whereby drivers are required to pay a fee to drive in certain city areas. London introduced congestion charges in 2003 and, more recently, the Ultra-Low Emission Zone, which imposes stricter emission standards. Similarly, Gothenburg (Sweden) has applied an electronic congestion tax since 2013, which reduced traffic by approximately 10% during peak hours and promoted the use of public transport (68). These policies encourage a shift away from private motorized vehicles toward more sustainable modes of transportation, including cycling.

Beyond traffic regulation, innovative urban planning models have been introduced to reduce car dependency. Paris has advanced the concept of the “15-min city,” where essential services are accessible within a short walk or bike ride, thereby reducing car trips and supporting active travel. Barcelona has developed superblocks—urban units that restrict through-traffic to prioritize pedestrians, cyclists, and social spaces—helping to expand greenery and mitigate heat island effects. Hamburg has gone even further, aiming to become a fully car-free city by 2035 to cut traffic, noise, and pollution while prioritizing active and public transportation (69).

These initiatives contribute to expanding green urban spaces, with vegetation filtering atmospheric PM and sequestering CO₂. Moreover, green spaces improve biodiversity, reduce heat islands, and promote citizens' physical and mental health (70, 71). Ultimately, such strategies align with the objectives of the European Green Deal, which seeks to achieve climate neutrality by 2050, positioning AC as a central component of healthier, more resilient, and sustainable urban environments.

### 2.3 Current state of active commuting in Europe: participation rates and barriers

According to a 2019 Eurobarometer survey, 8% of European Union citizens use bikes or scooters (including e-scooters, which are not considered active modes of transportation) as their primary mode of transport, with notable prevalence variation across countries, ranging from 41% in the Netherlands to 0% in Portugal (10). Overall, Mediterranean countries exhibit lower rates of cycling. A report by Interreg Europe in the same year indicated that approximately 20%–40% of all journeys in the European Union are performed by walking and cycling, although the purpose of travel is not explicitly referred to as commuting (72). Indeed, to the best of our knowledge, there is no data about the participation rates of AC.

**Figure 1:**
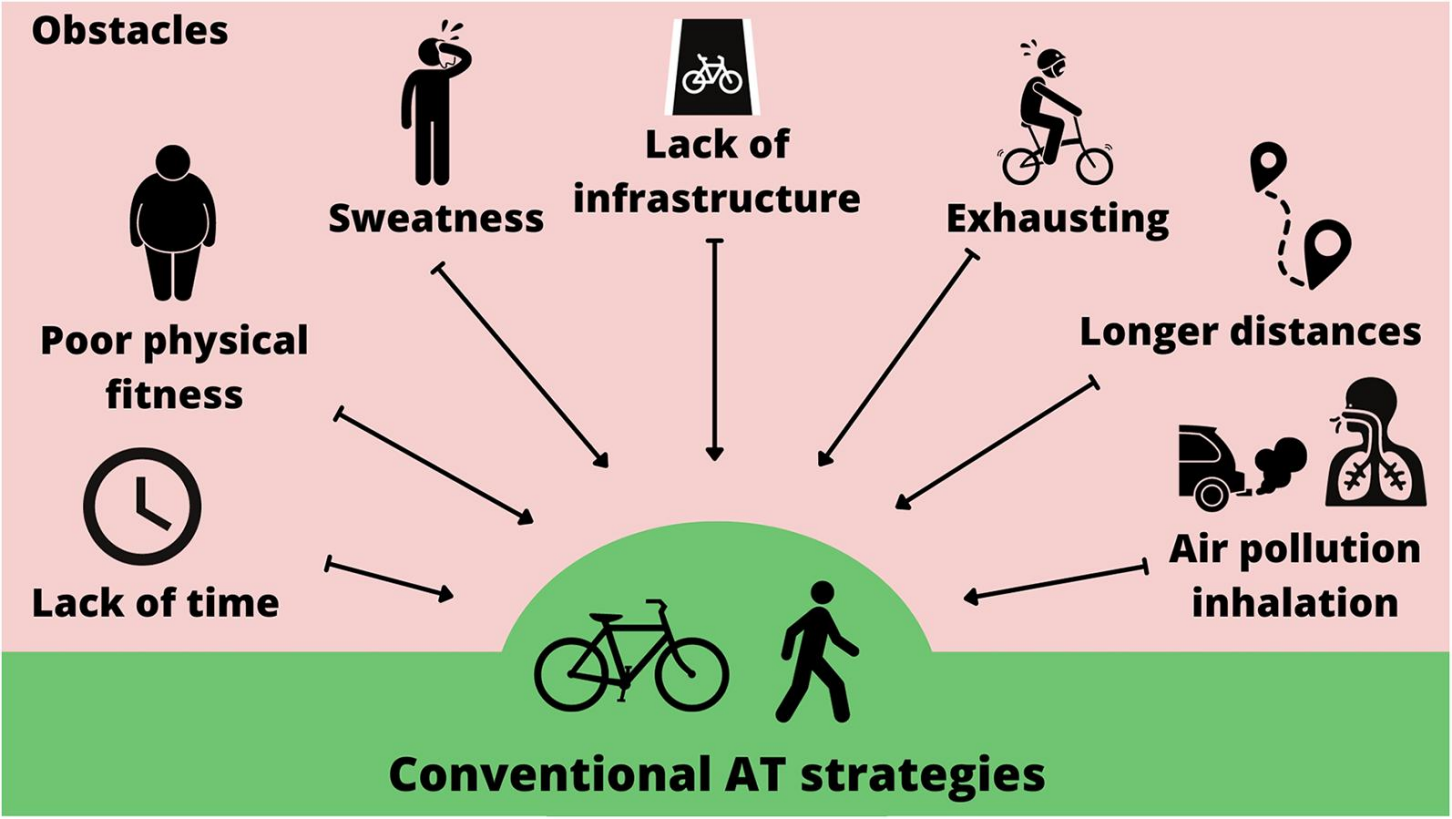
Representative barriers that discourage the adoption of conventional active transportation modes, including cycling and walking. AT, active transportation.

Published data indicate a substantial opportunity for European countries to promote AC, as over 50% of car trips are less than 3 km and 75% are less than 5 km, distances easily manageable by walking or cycling (73). Nevertheless, passive transportation remains predominant, with car use increasing by 18% in Europe between 2000 and 2019 (74), a trend reinforced by car-oriented infrastructure that continues to disincentivize AC and stimulate car dependence (27). Also, the characteristics of land use and public transportation availability are linked to active transportation (75). Several other factors contribute to the limited adoption of conventional AC: the presence of hilly terrains, poor physical fitness, a lack of time and distance to work, the feeling of sweating before working, inadequate infrastructure, and the absence of bike-sharing services (5, 6) (Figure 1). Furthermore, psychological factors may determine the intention to shift toward AC. According to the theory of planned behavior, intention is influenced by attitudes, perceived behavioral control, and subjective norms (76). These factors may influence AC adoption, as the intention is a strong predictor of behavior change, such as the shift to a sustainable travel mode (i.e., e-bike) (77). Therefore, overcoming the practical and psychological barriers to AC adoption is essential to harness its full potential—not only as a way to increase daily physical activity but also as a lever to support healthier, more sustainable, and less car-dependent urban environments.

## 3 The potential role of electric bikes

The term “e-bike” covers a wide range of electrically assisted bicycles. Pedelecs (i.e., pedal electric cycles) are e-bikes that provide motorized assistance only when the rider is pedaling. The motor provides a boost of up to 25 km/h, which makes pedaling more accessible and less exhausting. Unlike throttled e-bikes or e-scooters, which work without human propulsion, pedelecs require continuous physical input, classifying them as excellent active transportation options. For this review, the term e-bike refers specifically to pedelecs.

E-bikes could bypass some commonly reported barriers to cycling, such as the presence of hilly areas, time constraints, limited fitness levels, and long distances to work (78, 79), representing an excellent strategy for commuting (80, 81). Indeed, e-bikes allow users to travel at higher speeds with less effort than conventional bicycles (7), enabling participants to travel long distances and decreasing their travel time (6). This advantage is particularly relevant in modern societies where individuals face increasing time pressure and longer working hours (82). Moreover, the reduced physical effort also allows commuters to arrive at work less fatigued and sweaty, addressing concerns related to limitations imposed by inadequate workplace amenities, such as the absence of shower facilities, and the need to wear clean and appropriate clothing during the workday (83). Furthermore, e-bikes facilitate smoother and less stressful journeys within congested urban areas, where traditional car commuting can increase travel times and stress levels (84). By enabling more efficient journeys through traffic and reducing the time spent searching for parking, e-bikes allow workers to leave home at convenient times, arrive more relaxed at work, and better integrate commuting into busy schedules. Moreover, using e-bikes for commuting may also encourage additional cycling trips for other purposes, further increasing overall physical activity levels. Supporting this, a study conducted in Norway revealed that shifting to e-bikes increased the number of e-cycling trips up to 1.4 per day and the distance cycled up to 10.3 km (81). E-bikes are also employed for leisure activities and may provide an opportunity to promote cycling among older adults, people with overweight, and other subgroups who commonly experience challenges with engaging in regular physical activity (85, 86).

**Figure 2:**
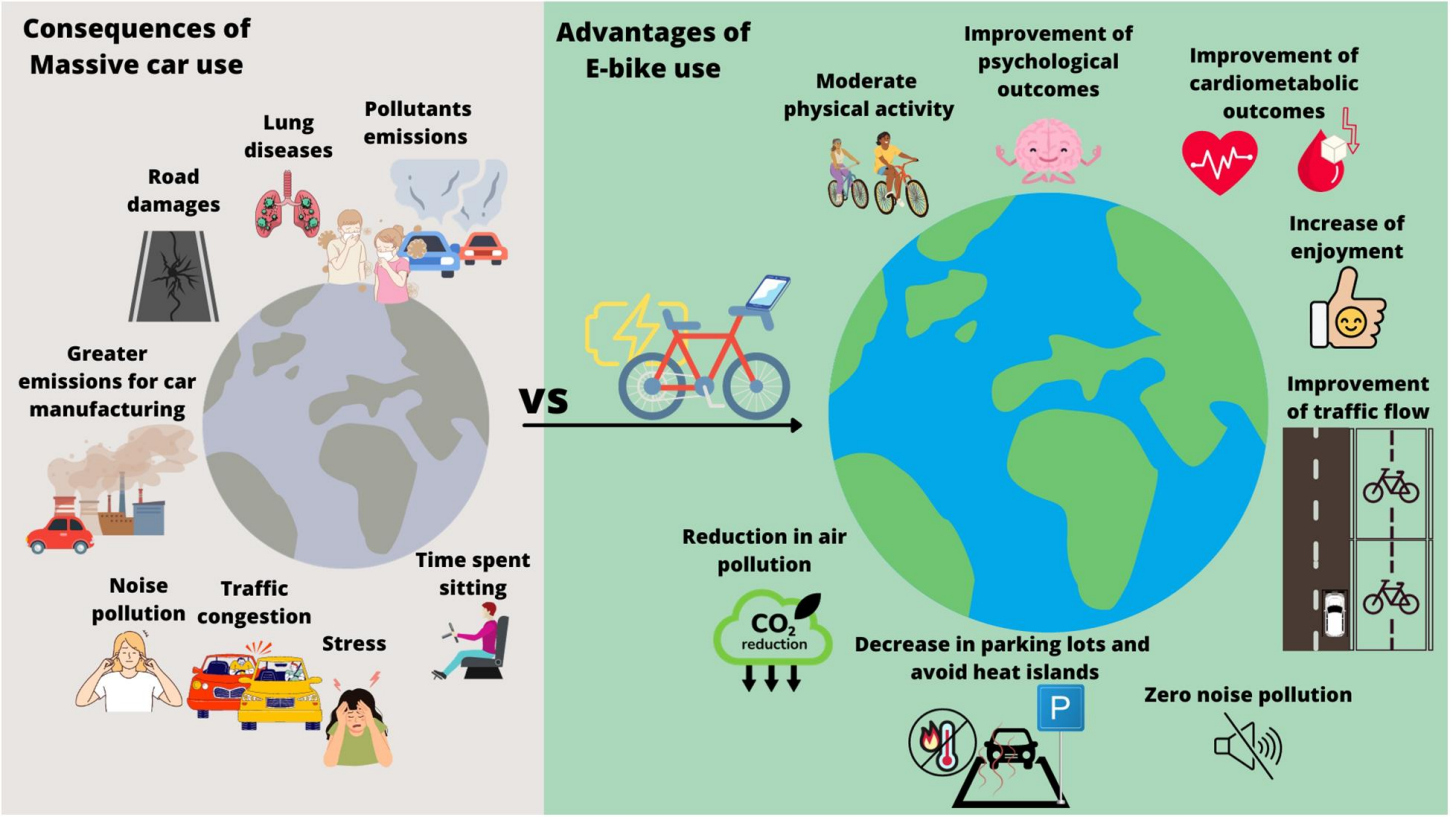
Comparison between the consequences of using motorized vehicles (on the left) and e-bikes (on the right).

Although pedaling with an e-bike is less intense than conventional cycling (4.1–6.1 METs vs. 6.4–8.2 METs) (7), research indicates that regular e-cycling yields several health outcomes, depending on the duration and intensity of physical activity (87). A randomized study performed in Switzerland revealed that after 4 weeks of e-cycling, the maximum power output and VO_2_ peak increased (86), which is highly clinically relevant because cardiorespiratory fitness is an essential risk factor for cardiovascular mortality (88). Additional studies noted improvements in other cardiometabolic risk factors: 4 weeks of e-cycling at least three times a week for 40 min per session, decreased 2-h plasma glucose during an oral glucose tolerance test (89). A study conducted in Britain revealed that people who shifted from passive to e-bike commuting reported better physical health, more productive organizational behavior, and more positive affect (90). Also, the level of enjoyment is greater for e-bikes than for conventional bikes, probably because of less perceived exertion (91), thus encouraging physical activity adherence. All the potential health advantages of e-bikes are shown in Figure 2.

Overall, these health-related findings suggest that e-bikes could benefit people affected by chronic pathologies, which require physical activity to manage the disease. However, many of these patients are deconditioned, have lower fitness levels, and thus may not sustain the fatigue of a conventional bicycle (92, 93). By reducing pedaling effort, e-bikes offer an innovative approach to encourage outdoor physical activity among these patients. Although research in this area is relatively new, studies on stroke survivors (93), diabetic patients (92), and breast cancer survivors (94) demonstrate the safety and effectiveness of e-bike use.

Alongside physical activity-related benefits, e-bikes represent an interesting way to promote green, sustainable transport capable of facing urban congestion and pollution problems (95, 96). Based on previous studies, the energy efficiency of e-bikes is estimated to be 18 times higher than that of cars (97). Indeed, unlike cars, e-bikes do not burn fossil fuels while pedaling (tank-to-wheel) as electric energy is employed. A modeling study conducted in England estimated that replacing private car trips with e-bike journeys may reduce CO_2_ by up to 24.4 million metric tons per year (95). Another research based on the Switzerland scenario found that greenhouse gas emissions may decrease by up to 10% when e-bikes are used for commuting (98). Moreover, a study performed among the staff of the National Health Service of the United Kingdom reported that switching to an e-bike for commuting generated a decrease of 34.3% in CO_2_ emissions and energy savings of 35% per person annually (99). Further study in Sweden revealed that a range between 14% and 20% reduction in emissions of CO_2_ is estimated when at least 52% of the car journeys are substituted by e-bike (100).

Although the energy required to manufacture an e-bike is significantly lower than that required to build a car (27), it is important to highlight that e-bikes are not completely emission-free, as battery production (well-to-wheel), maintenance, and disposal generate carbon emissions (64). For example, producing lithium-ion batteries requires critical raw materials such as lithium, cobalt, and nickel, whose extraction is energy-intensive and often associated with environmental concerns (101). Nonetheless, the entire life cycle carbon footprint of e-bikes is far lower than that of vehicles with internal combustion engines (102) and about two times lower than e-cars (103). Modeling studies indicate that, over their entire life cycle, electric vehicles generate only about 37% of the CO₂ emissions produced by fuel-powered vehicles, with results strongly influenced by the energy mix used for power generation (e.g., renewable sources vs. coal-fired plants) (104). Although e-bikes take us closer to greener transportation, more effort is needed to minimize their ecological footprint. In this regard, the implementation of solar-powered charging stations offers a promising pathway to further lower the environmental impact of e-bike charging.

Beyond their quasi-eco-friendly nature, e-cycling also offers potential benefits for urban mobility (Figure 2). These advantages encompass various aspects of city life:

a. Noise pollution reduction: unlike motorized vehicles, e-bikes do not emit any sounds, ensuring an effective solution to noise pollution (105). Thus, by encouraging more people to commute *via* e-bikes, cities can experience quieter streets and a more pleasant environment.
b. Traffic congestion relief: e-bikes take up significantly less road space than cars, contributing to reduced traffic congestion (37, 105). If more commuters use e-bikes for shorter trips, it would improve traffic flow, making travel faster also for those who rely only on motorized vehicles due to longer distances.
c. Road preservation: e-bikes are lightweight and thus are less likely to cause road surface damage, reducing maintenance costs and ensuring safer roadways.
d. Parking lots reduction: traditional parking lots require energy-intensive materials like asphalt, which contribute to urban heat islands and pollution (106). Heat accumulation in open-air parking lots increases the local temperature, contributing to the global warming crisis. The promotion of e-bikes and AC, in general, may reduce the need to construct new parking spaces, mitigating the formation of additional urban heat islands (5).

### 3.1 Challenges and strategies for e-bike adoption: the role of policy and technology

In Europe, e-bikes sales grew by approximately 30% annually from 2006 to 2017 (59). In 2024, around 6.68 million e-bikes were sold, with Germany leading the market at 2.1 million units sold, followed by France, the Netherlands, and Italy (107). Although the rising awareness of environmental and physical inactivity contributed to this expansion, economic factors have also played a significant role. A recent survey conducted across 12 European countries found that the rise in fuel prices and cost of living was among the primary motivators for individuals to consider buying and using e-bikes (108).

Despite steady growth, the European e-bike market remains slower and more fragmented than in China, which stands as the most advanced globally. One of the main reasons may be the higher purchase price of e-bikes in Europe, averaging €2,242, the highest worldwide, compared with a global average of €711 and just €373 in Asia. Even within Europe, notable disparities exist, with prices in Central and Western Europe being almost four times higher than in Eastern Europe (107). It is also important to note that these statistics include all e-bike categories, such as road and off-road bicycles, while excluding shared mobility services.

In China, the rapid growth of sales has directly translated into widespread e-bike use, gradually replacing not only private cars but also large shares of public transportation trips (109). A similar trend has been observed in Australia (110) and North America (111), where e-bikes are increasingly perceived as a practical alternative to car trips. By contrast, in countries with traditionally high cycling rates, such as the Netherlands, e-bikes are often replacing conventional bicycles rather than cars, suggesting that their role depends strongly on the cultural and infrastructural context.

Despite the market expansion and the plethora of health and environmental benefits associated with e-bike use, multiple concerns and barriers still hinder widespread adoption across different regions of Europe. Several barriers contribute to this situation, which can be broadly categorized as economic, practical, infrastructural, and social.

The upfront purchase price of an e-bike is well recognized to be the primary barrier to e-bike adoption, as it discourages many potential users. To overcome this challenge, the role of companies and policymakers is crucial. Financial incentives, such as subsidies for e-bike purchases, have proven to be among the most effective strategies to improve accessibility (112). A recent review highlighted that immediate incentives, particularly point-of-sale discounts, are the strongest drivers of consumer behavior, followed by tax deductions and delayed mechanisms such as mail-in rebates (113).

Across Europe, there are several initiatives that help with the purchase of an e-bike. In the United Kingdom, the Cycle to Work Scheme allows employees to purchase an e-bike with discounts of up to 47% while spreading payments directly through salary deductions, thus reducing the upfront burden (114). Similarly, in Vienna and Italy, purchase bonuses of up to 50% of the retail price were introduced in 2024 (115). Portugal became the first European country to apply a reduced VAT (from 23% to 6%) rate for bicycle and e-bike purchases in 2023 (116).

Alongside purchase subsidies, financial rewards are a noteworthy strategy. Research conducted in the Netherlands demonstrated that monetary incentives ranging from €0.08 to €0.15 per kilometer travelled by e-bike over one year significantly stimulated a modal shift from cars to e-bikes, and e-bike travel increased by up to 73% after just six months of participation (97). However, further research is needed to determine whether travel behaviours remain consistent once incentives are withdrawn or if users revert to their previous commuting modes.

Insufficient infrastructure or difficulties accessing it represent a major concern regardless of the type of bicycle used (6, 117). Specifically, the lack of cycling lanes and the presence of poor road conditions (e.g., potholes and uneven surfaces) have been extensively documented (6, 117). The lack of charging infrastructure poses significant challenges to the growth of electric vehicles in Europe (118), including e-bike adoption. Even in countries where e-bikes are emerging, insufficient charging points, especially in the workplace, reveal a crucial infrastructural gap that must be addressed. Additionally, the lack of safe parking lots for e-bikes represents a critical issue, as most people indicate that the risk of theft is a significant concern in e-bike use (117).

To address these constraints, municipalities and companies must collaborate to make an e-bike–friendly environment. One starting point is the extension of cycle networks that allow linking residential areas to key destinations such as the workplace or commercial districts. As well, ensuring protected lanes, such as using barriers or parked cars as buffers between cyclists and moving traffic, may minimize the risk of injury. Charging facilities should be strategically placed in the workplace or near bus stops to facilitate easy switching between different modes of sustainable transport. In parallel, secure monitored parking lots would reduce theft-related concerns and further encourage adoption. Finally, expanding e-bike sharing systems may represent an additional strategy to reduce upfront purchase costs, particularly in urban contexts (119).

In addition to practical barriers, social stigmatization and perception limit the appeal of e-bikes (117). In different surveys, people considered e-cycling because it requires less physical effort than conventional cycling (111, 120, 121). This impression condemns the marketing of e-bikes as an authentic mode of AC and discourages potential users from considering them a daily alternative to motorized transport. Thus, awareness campaigns or trial periods may be effective strategies to partially address this skepticism. Indeed, several studies based on the theory of planned behavior suggest that individuals are more likely to adopt e-bikes when they perceive them as useful (e.g., convenient, cost-effective, and easy to use) and when colleagues or friends also use them and support their use (121, 122).

One effective intervention is offering free e-bikes to individuals for a limited time, a practice already common in the USA, Norway, and the Netherlands. For example, a study conducted at Delft University (Netherlands) showed that after a trial period, car use for commuting decreased from 88% to 63%, while e-bike use rose from 2% to 18% (123). Similar findings were reported in Switzerland, where participants reduced car dependency not only if they purchased an e-bike but also if they did not, demonstrating the long-term influence of trial exposure (124).

Workplaces offer another important setting for promoting e-bike commuting. Employees who are supported by colleagues in purchasing or using e-bikes or are exposed to a culture of sustainable commuting within their company are inclined to adopt this mode of transport (122, 125). This is a win–win situation where employees may improve personal health through AC, whereas employers may gain from higher productivity and reduced absenteeism at work (58). Also, encouraging sustainable commuting may enhance the company's public image, showing commitment to environmental responsibility. To foster this cultural shift, companies should not only provide financial incentives but also invest in infrastructure facilitations, including safe parking lots and workplace charging stations (126).

Another significant barrier is safety related to e-bike use. Several people are afraid of being severely injured in the case of an accident because of the higher speed compared with conventional bikes (117). Indeed, research conducted in Switzerland revealed a higher frequency of traffic accidents among e-bikers than conventional bikers (127). Several studies have identified speed as a key factor contributing to the high incidence of e-bike crashes, as increased cycling speed affects riders' behavior and reduces their ability to anticipate movements in traffic (128). Moreover, much of the existing cycling infrastructure is not designed to accommodate the higher speeds of e-bikes and sometimes allows pedestrians to walk on bike lanes. However, responsibility for safety does not lie solely with e-bike users; it also depends on the education of motor vehicle drivers, who should ensure the protection of vulnerable road users, including both cyclists and pedestrians (128). For this reason, it is essential to promote a culture of cycling not only among cyclists, who must continue to follow traffic rules, but also among drivers of other vehicles, who need to be aware of the potential presence of bicycles.

In addition to these measures, advances in intelligent transportation systems and modern technology provide opportunities to further enhance cyclist safety (129). For instance, municipalities could implement targeted solutions such as traffic lights that give cyclists a few seconds of green before motor vehicles, improving visibility and reducing the risk of collisions, particularly with turning cars.

Over the past decades, advanced rider assistance systems have been developed to address these safety concerns. For instance, despite not being designed for e-bikes, Xie and colleagues developed sensors that detect vehicles or obstacles around cyclists and emit alerts to help prevent collisions (130). Another risk is the potential for falls when suddenly braking on an e-bike, often due to loss of stability or rear-wheel lift. To address this concern, Mayer and colleagues have developed and tested an anti-lock braking system designed for e-bikes to prevent front-wheel lock and reduce the risk of rollover (131). A commercial version of this type of system has already been implemented, such as Bosch's e-bike which regulates braking pressure and enhances stability during sudden braking (132).

In addition to safety-focused innovations, advances in intelligent transport science have also sought to improve not only the overall riding experience and comfort, aiming to make e-cycling more accepted but also to transform the e-bike as a tool to monitor air pollution levels.

To this scope, several types of sensors and smart systems are increasingly used to monitor real-time data during cycling. Common sensors include the global positioning system (GPS), power sensors to measure pedaling force, heart rate monitor, and speed and cadence sensors (133). For instance, Giani and collaborators designed a smart e-bike that allows an automatic change of the electrical assistance level to maintain a constant physical effort based on heart rate sensors (134). Tandon and colleagues developed an intelligent system that adjusts pedaling cadence automatically, keeping it within a comfortable range (135). Thus, when riders suddenly change their cadence due to a specific condition (e.g., hilly terrain), the monitoring system automatically adjusts the gear and provides more assistance to help reset the preferred cadence. However, further studies should evaluate the user acceptance of these smart e-bikes and the health benefits related to their use compared to traditional e-bikes.

Other researchers have focused on the collection of traffic and air pollution data. For instance, Andres and colleagues designed “Ari”, a smart e-bike that automatically adjusts speed based on location and timing data in order to avoid red traffic lights, facilitating smoother journeys (136). Aguiari and colleagues designed an e-bike equipped with a sensor called Canarin II, capable of real-time and street-by-street monitoring of PM levels at the urban level (137). Monitoring traffic and air pollution through e-bike–embedded sensors could have important practical implications. Municipalities could leverage such technologies, particularly within shared mobility schemes, to map and gather environmental pollution using Internet of Things technologies, optimizing urban mobility, and supporting policies aimed at creating healthier, more sustainable, and data-driven smart cities. From the user's perspective, integrating pollution data directly into the riding system could help cyclists avoid highly congested or polluted areas by suggesting alternative routes or by automatically adjusting motor assistance to reduce effort and, consequently, pollutant inhalation. In line with this perspective, we proposed an ambitious prototype of an e-bike that offered personalized motor assistance under different conditions, such as fatigue, traffic, and air pollution (138). However, further studies should attempt to establish the feasibility of the algorithm used to integrate all the parameters involved and minimize the costs associated with such sensors and the weight of the e-bike.

In light of the different natures of the barriers (i.e., practical and social), offering a transdisciplinary and collaborative approach to limit them is essential. Indeed, e-bikes could play an important role in the coming years, as the European Commission proposed banning the sale of fossil-fueled cars from 2035 and becoming climate-neutral from 2050. Hence, e-bikes could help achieve the “Fit for 55” package, which aims to reduce pollutant emissions by 55% by 2030 (139). This challenging scenario forces multiple stakeholders and experts beyond public health to collaborate to produce technological and infrastructural progress and change people's behavior. Thus, employers, psychologists, urban planners, educators, engineers, and exercise specialists should collaborate to create a greener and more physically activity-oriented social community. To do this, it will be necessary to take action in workplaces, schools, and transportation infrastructure. New, safe, and more accessible cycle paths, along with strategies to make cycling enjoyable and effortless, as well as education for patients, clients, and youth about the risks associated with a sedentary lifestyle (including over-reliance on motorized vehicles), are interventions worth reinforcing.

## 4 Conclusions

Physical inactivity, sedentary behavior, and air pollution remain the major health challenges of this century in Europe. The increasing prevalence of chronic diseases associated with inactive lifestyles, combined with current concerns about climate change, requires more actions to create more sustainable and active living. By incorporating AC into daily routines, there is more chance to be physically active and reduce the sedentary behavior associated with car driving. Specifically, e-bikes may bypass the common barriers to active transportation, providing an effective alternative to motorized transport to commute.

The current review highlights the combined potential of e-bikes to address physical inactivity, sedentary behavior, and air pollution simultaneously, improving overall health and urban mobility. Specifically, e-bikes may bypass common barriers to active transportation, providing an effective alternative to motorized commuting. However, several barriers still hinder e-bike adoption for commuting. This review has summarized the main obstacles, including economic factors, practical concerns such as the lack of safe cycling infrastructure, and social perceptions. Corresponding solutions and best practices across European regions have been discussed, alongside technological advancements that enhance e-cycling safety and enable e-bikes to function as devices for environmental data collection, such as air pollution and traffic monitoring. However, further research is needed to determine whether these strategies can lead to a sustained shift from motorized vehicles to AC, specifically e-bike use, and to assess their long-term impacts on public health and the environment.

It should also be noted that while studies on e-bikes exist, many adopt a general transport perspective, encompassing multiple purposes (e.g., leisure activities). Since commuting is a daily activity with specific requirements, such as time constraints, there is a clear need for more targeted research focusing exclusively on commuting purposes.

Finally, substantial regional differences in e-bike adoption exist not only in comparison with China, which represents the leading global market, but also within Europe, with Northern countries, particularly the Netherlands, being among the most advanced. This helps explain why most research has been conducted in Northern European countries, leaving limited knowledge of the factors affecting e-bike use in other regions. Thus, future research should also focus on Mediterranean European countries, where levels of AC are generally lower, to better understand regional barriers and opportunities for promoting AC and e-bike adoption.

To conclude, we acknowledge the limitations of this review. First, the selection of articles may have been influenced by some degree of author bias. Second, this review focused on studies retrieved only from Google Scholar and PubMed, which may have resulted in the exclusion of relevant research available in other databases, such as Scopus. These limitations should be considered when interpreting the findings and highlight the need for broader systematic investigations in the future.
